# Brain Tumour Temporal Monitoring of Interval Change Using Digital Image Subtraction Technique

**DOI:** 10.3389/fpubh.2021.752509

**Published:** 2021-09-21

**Authors:** Azira Khalil, Aisyah Rahimi, Aida Luthfi, Muhammad Mokhzaini Azizan, Suresh Chandra Satapathy, Khairunnisa Hasikin, Khin Wee Lai

**Affiliations:** ^1^Faculty of Science and Technology, Universiti Sains Islam Malaysia, Bandar Baru Nilai, Malaysia; ^2^Department of Electrical and Electronic Engineering, Faculty of Engineering and Built Environment, Universiti Sains Islam Malaysia, Bandar Baru Nilai, Malaysia; ^3^School of Computer Engineering, Kalinga Institute of Industrial Technology, Deemed to Be University, Bhubaneshwar, India; ^4^Biomedical Engineering Department, Faculty of Engineering, Universiti Malaya, Kuala Lumpur, Malaysia

**Keywords:** brain imaging, image subtraction, interval change, tumour progression, magnetic imaging resonance

## Abstract

A process that involves the registration of two brain Magnetic Resonance Imaging (MRI) acquisitions is proposed for the subtraction between previous and current images at two different follow-up (FU) time points. Brain tumours can be non-cancerous (benign) or cancerous (malignant). Treatment choices for these conditions rely on the type of brain tumour as well as its size and location. Brain cancer is a fast-spreading tumour that must be treated in time. MRI is commonly used in the detection of early signs of abnormality in the brain area because it provides clear details. Abnormalities include the presence of cysts, haematomas or tumour cells. A sequence of images can be used to detect the progression of such abnormalities. A previous study on conventional (CONV) visual reading reported low accuracy and speed in the early detection of abnormalities, specifically in brain images. It can affect the proper diagnosis and treatment of the patient. A digital subtraction technique that involves two images acquired at two interval time points and their subtraction for the detection of the progression of abnormalities in the brain image was proposed in this study. MRI datasets of five patients, including a series of brain images, were retrieved retrospectively in this study. All methods were carried out using the MATLAB programming platform. ROI volume and diameter for both regions were recorded to analyse progression details, location, shape variations and size alteration of tumours. This study promotes the use of digital subtraction techniques on brain MRIs to track any abnormality and achieve early diagnosis and accuracy whilst reducing reading time. Thus, improving the diagnostic information for physicians can enhance the treatment plan for patients.

## Introduction

Malignant brain tumours are prevalent, with more than 500 new cases diagnosed daily worldwide (1). Tumour grades (I–IV) depend on the location and how easily cells replicate and migrate to other vital organs (2). In most cases, magnetic resonance imaging (MRI) is used to diagnose this tumour (MRI). The use of intravenous (IV) gadolinium-enhanced MRI to help provide a more definite picture of a brain tumour is common (3). This is when a patient initially undergoes a standard MRI and then receives a particular form of a contrast material called gadolinium via IV (4). The dye is then used in a second MRI to obtain another series of images. The procedure of diagnosing a tumour, on the other hand, takes a lengthy time. Therefore, early detection or abnormality progression of tumours is needed to prevent its migration to other organs (5). Early diagnosis is also essential to prevent patients from suffering a severe stage of the disease. A system that involves at least two input images in generating a digital subtraction image. The device is divided into registration subsystems according to different values of registration parameters to improve the registration of various input images (6). According to the pixel value of or around the related pixel positions of subtracted pictures, the combined subsystem is then arranged to incorporate pixel values to positions of merged image pixels (7). The application of digital image subtraction techniques for temporally and spatially sequential brain images is investigated in this study using brain magnetic resonance imaging (MRI) to distinguish interval changes and provide radiologists with early notification to improve their diagnostic accuracy.

Various medical imaging procedures, such as CT, MRI and ultrasound scans, are available. The entire scan will be diverse in time processing (8). The time of operation depends on the area of the scanned body (9, 10). Analysis and reading images using CT machines takes ~5–15 min because these scans use radiation for a few hours before the test compared with using contrast dye, which prohibits patients from eating or drinking (11). The ultrasound is estimated to take ~45 min (12). However, helical CT, which was implemented in 2003, was used to achieve this from the point of view of early diagnosis; however, helical CT is an inconvenient screening tool that takes a considerable amount of time for interpretation (13). MRI scans use powerful magnetic fields and radio wave pulses to create cross-sectional images of the body that typically take approximately an hour, on average, from start to finish (14). Meanwhile, the current processing time of the image subtraction method for chest images is long at ~12 min for an image pair (15, 16). However, using a fast computer and optimising the software may reduce the processing and reading time as well as detect abnormalities with high sensitivity.

The proposed digital image subtraction technique can help track any interval changes or abnormality and achieve early diagnosis of brain tumours (17, 18). Image subtraction on various commercial systems is accessible. In order to execute image subtraction, unenhanced and enhanced T1-weighted pulse sequences should be maintained consistently in all image parameters, including receiver gain and image scale factor (19). Doctors and radiologists have only been using manual methods for observing the interval change of brain images. The statistics showed that false-positive results are increasing every day (20). New agents, such as antiangiogenic agents and immunomodulators, can cause the appearance of waxing and waning lesions (21). Moreover, a central reviewer may be blinded in terms of the temporal sequence (22). This simple digital technique can help obtain fast and accurate results without requiring any extra testing to confirm the results. Thus, improving the diagnostic information of physicians can help increase the spatial resolution of MRI regardless of the slice thickness, RBW and matrix for creating an efficient and effective treatment plan for patients (23). Precision is needed because it can affect the powering of clinical trials (24). The sample size is related to both the effect size and variance of the outcome measurement. MATLAB is the programming software used in the process of this study.

A grading scheme is often used for adult brain tumours because it provides characteristics that help radiologists decide on the severity level of the cancer and its likelihood in developing in any part of the brain (25). A higher grade is assigned to tumours with features typically associated with faster growth, whereas a lower grade typically indicates a more positive prognosis for patients (26). Several variables determine and help doctors decide on the optimal brain tumour treatment plan and patient prognosis. The tumour type and grade must be defined in determining the optimal treatment for brain tumours (27). Doctors consider both variables to understand how the tumour will respond and help assess medication choices. Convolutional neural network is a machine learning algorithm that has been successfully utilised in the segmentation and classification of images without using invasive measures (28). A T1-weighted contrast-enhanced MRI image database comprising three types of tumours was used in a previous study to perform classification without pre-processing (29, 30). However, the generalisability of this technique must be improved in classifying and accurately locating tumours. Two studies performed the same longitudinal imaging algorithm in patients with multiple sclerosis; the results showed a substantial reduction in reading time compared with standard assessments and increased diagnostic precision for lesion identification (25, 31, 32). The digital image subtraction technique allows tumours or other abnormalities to be tractable with a simple algorithm (33). Manual registration will require more time to diagnose the diseases compared to auto-subtracted registration. In clinical practise with a strong clinical impact and a solid inter-observer agreement, multimodality is already currently conceivable. However, we have proposed the image subtraction of the MR image in order to analyse the tumour growth within the brain. It is the most extensive application for a change detection method and can be used in a wide range of image types and geographical environments. The current technique that has been used is the manual conventional registration reading, which is only by personnel estimation. While, the proposed method, which is the auto-subtracted registration technique, will provide more reliable treatment planning and less time-consuming as to track abnormalities changes on MRI images.

Therefore, the digital image subtraction technique is used in this study for detecting abnormalities in brain images to solve these problems. This study aims to (1) develop a temporal digital image subtraction technique using MATLAB, (2) establish spatial sequential digital image subtraction techniques using MATLAB and (3) test the accuracy of algorithms on five sets of brain MRIs.

## Materials and Methods

This retrospective study was performed after obtaining prior approval from the Medical Research and Ethics Committee (MREC) and the ethics committee of Universiti Sains Islam Malaysia (USIM). All images were accessed anonymously from an online database and informed consent from patients was unnecessary because data are publicly available for educational purposes, as outlined in Creative Commons Attribution 3.0 Unported Licence (34). Five pairs of conventional brain images, including the number of distortions, misrepresented cases and appropriately represented cases, were acquired from five different patients. Five original images were obtained before treatment, and another five current images were obtained after treatment. MATLAB was used to apply the proposed technique. Images were resized to a 512 × 512 matrix dimension for temporal and spatial subtraction.

Firstly, five pairs of subtraction image sets were classified into three groups. Groups A, B, and C consist of current, original, and subtracted images, respectively. Image subtractions in Groups A and B must be indifferent to the time interval between each other. The results of image subtraction classified as C were the critical focus cases used in this study to develop and assess the current initial registration scheme. The development program MATLAB was applied to generate these temporal subtraction images. The effect of the temporal subtraction image was evaluated using an observer performance study and receiver operating characteristic analysis. The computer-aided diagnosis (CAD) technique consists of temporal and spatial parts (35).

The following methods are used in this study. Images were inputted essentially with a template region of interest (ROI) in the search (current). Selected template (original) images demonstrated remarkably higher current ROI than the original ROI (36). The cross-correlation value between the original region in one image and the plural subregion at a different position within the current area ROI of another image was determined. Causes of radiographic misregistration were classified into four types, namely, brain expansion, lateral inclination, inclination and rotation (16). Therefore, non-linear distortion (warping) of an image relative to another was required to obtain an accurate registration.

Temporal transformation (time interval difference mode) consisting of the three steps of the temporal CAD program (global registration, regional registration, and features) was conducted. This technique is a time-manipulated process and closely related to simple mask subtraction, in which an early image is chosen as the mask and single masks are subtracted from each consecutive image of the series to form a series of subtracted images. The image acquisition process in this study begins 5 s after the injection at a rate of five images per second. Five image sets were obtained from five different patients. These image pairs were acquired at two different follow-up (FU) time points ranging from 0.6 to 3 months.

Spatial transformation (space interval difference mode) related to the space distribution was also performed to describe the spatial part. This determination started with preprocessing an image. Pixel level of the size-detection algorithm was applied to detect suspicious locations, followed by segmentation of suspicious locations and then extraction of functionality. Thresholding method was implemented as follows (37):


(1) $$\begin{array}{l} T_{1}=\frac{h_{1}+h_{2}}{2} \end{array}$$

(2) $$\begin{array}{l} h\left(i,j\right)=\{\begin{array}{c} 1\;if\;f\left(i,j\right)\geq T_{F}\\ 0\text{ if }f\left(i,j\right)<T_{F} \end{array}\\ if||T-T_{1}||\geq \Delta T \end{array}$$

where *T* is the new threshold value; *h*_1_ and *h*_2_ are mean intensities of *H*_1_ and *H*_2_, respectively; *h*_1_ is the original greyscale image that depends on intensity values; *h*_2_ is *f*(*i, j*); the initial threshold value *T* is selected, and *T*_*F*_ is the final threshold value.

The temporal segmentation of ROI that links the location and feature extraction for original and current regions for both temporal and spatial CAD methods was classified. The ROI volume and diameter for both regions were recorded. Five temporal features were obtained by subtracting the original image values from the current image values. Another subtraction image obtained by merely shifting one of the images relative to the other indicated significant artefacts that obscure anatomic information. Shift values *x* and *y* were determined for a given set of x and y using a local template matching technique (38). Images were increasingly rotated by an angle α at the centre point (*x*_*C*_,*y*_*C*_) with the radical distance *r* reaching *r*_max_. This method indicated the interval change or any abnormality. Highly suspicious locations on the brain image were subdivided and features were calculated when selected and linked to their original locations. A non-linear warping technique was applied at the twirled (*x, y*) location for accurate registration of these brain images as follows (16, 39).


(3) $$\begin{array}{l} T_{X}^{-1}\;:x\;=\;\{\begin{array}{c} x_{C}+r\;.\;cos(\beta )\;for\;r\leq r_{\max}\\ \chi^{\prime }\;for\;r>r_{\max} \end{array}\\ T_{y}^{-1}\;:y\;=\{\begin{array}{c} y_{C}+r\;.\;sin(\beta )\;for\;r\leq r_{\max}\\ y^{\prime }\;for\;r>r_{\max} \end{array} \end{array}$$

with


(4) $$\begin{array}{l} d_{x}\;=\;\chi^{\prime }-x_{c} \end{array}$$

$$\begin{array}{l} r=\sqrt{d_{x}^{2}+d_{y}^{2}} \end{array}$$

(5) $$\begin{array}{l} d_{y}\;=\;y^{\prime }-y_{c} \end{array}$$

$$\begin{array}{l} \beta \;=\;Arctan\left(d_{y},d_{x}\right)+\alpha .\;\left(\frac{r_{\max}-\;r}{r_{\max}}\;\right). \end{array}$$

A volume and diameter error factor were estimated on the basis of the histogram analysis of brain images. A proper look-up table for the correction factor was chosen for non-linear density correction [Equations (6) and (7)] of improperly exposed radiographs. The ROI-SART algorithm can be expressed as follows (40):


(6) $$\begin{array}{l} l_{G}=\;p_{end}-\;p_{start},\;l_{V}=\overset{N}{\underset{n=1}{\sum }}w_{n},\;k^{ROI}=\frac{l_{V}}{l_{G}} \end{array}$$

(7) $$\begin{array}{l} {v_{j}}^{\left(k+1\right)}={v_{j}}^{\left(k\right)}+\lambda \frac{\sum_{{pi}_{\in }^{i}P\phi }\left(\frac{{k_{i}}^{ROI}p_{i}-\sum_{w_{in\neq 0}}^{N}n=1w_{in}{v_{n}}^{\left(k\right)}}{l_{G_{i}}}\right)w_{ij}}{\sum_{{pi}_{\in }^{i}P\phi }w_{ij}k_{i}^{ROI}} \end{array}$$

where *l*_*V*_ is the ray's length within the volume itself, *l*_*G*_ is the ray's length within the real ROI, *w*_*ij*_ is the entry of the matrix *W* that contributes to the entire ray *p*_*i*_, $v_{j}^{\left(k\right)}$ is the value of voxel *j*, l is the factor of relaxation and *P* is the angle φ projection. A volume composed of *N* cubic voxels *j* with constant values *v*_*j*_ is indicated as *v* and *p* is the vector of measured rays with the ray number *p*_*i*_ that goes through v.

**Figure 1:**
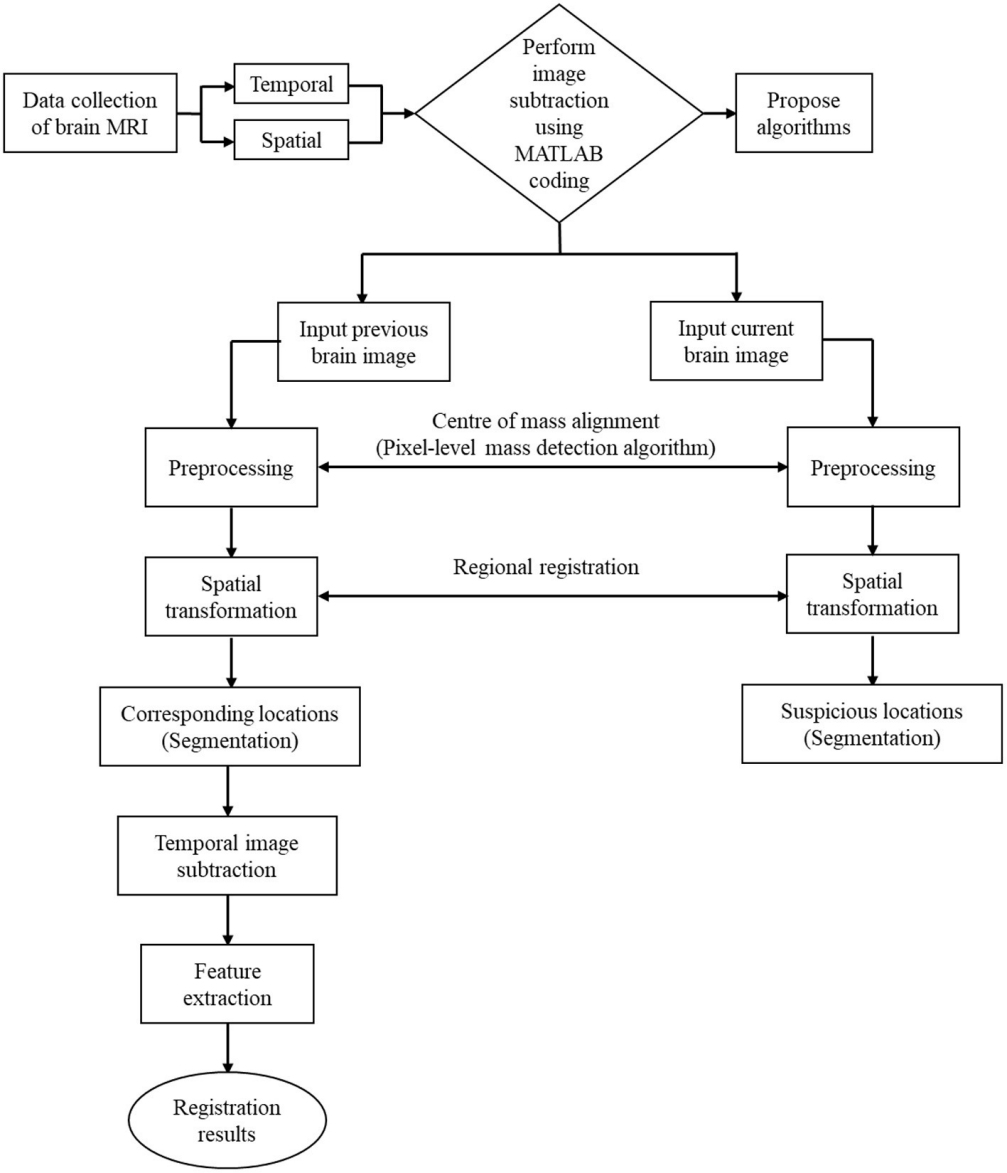
Overall process of image subtraction.

The output results obtained were indicated C images (subtracted images). Subtraction images were obtained by differentiating the original image from the warped current image. Contrast enhancement was applied to these result images. These subtracted images are two-dimensional (2D) enhancements calculated serially on post-contrast T1-weighted images and fluid-attenuated inversion recovery (FLAIR) hyperintensity volume to show the interval change or the presence of abnormalities that can indicate the progress of the disease. The result validation includes qualitative (experts' validation) and quantitative analyses (using root mean square error [RMSE]) (41). Experts (medical physicist and board-certified radiologists with experience in neuroradiology) separately conducted the conventional manual reading (gold standard). The gold standard reading was performed to distinguish between signal alterations of tumour progression and assess the existence of abnormalities and alterations related to posttreatment entities, such as pseudoresponse, pseudoprogression and radiation necrosis (RN). Figure 1 shows the overall outline of the complete method.

## Results

### Patient Cohort

Five patients (mean age: 48.2 ± 18.3 years, age range: 27–66 years and male) with position head first supine (HFS) were selected. All patients were diagnosed with abnormal cell growth [WHO grades I–IV (32)] and contributed five pairs (original and current images) of brain MRIs (Table 1). Patient 3 was diagnosed with pineoblastoma Grade I, which develops slowly and is unlikely to spread. Surgery can help the astrocytoma Grade II tumour in Patient 1. These cancers are unlikely to develop but are likely to return after surgery. Ependymoma Grade III tumours from Patient 2 and neurilemmoma Grade III from Patient 4 are likely to bear cells that differentiate rapidly and expand easily but without dead cells. Patient 5 was diagnosed with a malignant rhabdoid Grade IV tumour that exhibits rapid growth. Tumour location (42) and acquisition duration are listed in Table 1.

**Table 1:** Cohort characteristics of five patients.

**Patients**					
Cohort characteristics	1	2	3	4	5
Tumour Entity	WHO grade II	WHO grade III	WHO grade I	WHO grade III	WHO grade IV
Types of brain tumours	Astrocytoma (cyst)	Ependymoma	Pineoblastoma	Neurilemmoma	Rhabdoid tumour
Tumour location	Left hemisphere (Lobus frontalis)	Left hemisphere	Supraseller	Left hemisphere (Lobus frontalis)	Multifocal/Corpus callosum
Time since first diagnosis (months)	1	1	3	1	0.6
Substance of (last) CTX	Temozolomide	No	No	Bevacizumab	RTX
Additional patient history	CA	CA	CA	FU Tumour	FU Tumour
Slice thickness, location (mm)	5.00, 14.17	6.00, −56.16	5.00, 20.00	5.00, 22.31	6.00, 39.68
Age	30	58	27	60	66
Repetition time	450	3350	76.5	466.664	466.664
Acquisition duration	1.808*e*^+08^	1.746*e*^+08^	3.166*e*^+07^	1.596*e*^+08^	1.596*e*^+08^

### Evaluation of Treated Brain Tumours

#### Progress of Interval Changes

Table 2 shows five monitory patient cases with developing abnormality signals. According to gold standard readings, Patient 1 exhibited regression in tumour size, whilst patients 2, 4, and 5 showed progression in tumour size. Patients 3 and 5 exhibited a difference in abnormality location (mobility). All patients demonstrated changes in the shape of abnormalities.

**Table 2:** Results of registration on five patients.

**Patient**	**Planes**	**Current image**	**Original image**	**Subtracted image**
Patient 1	Axial	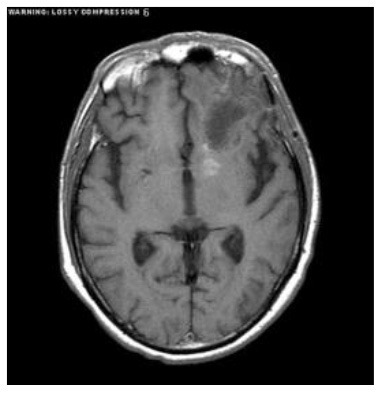	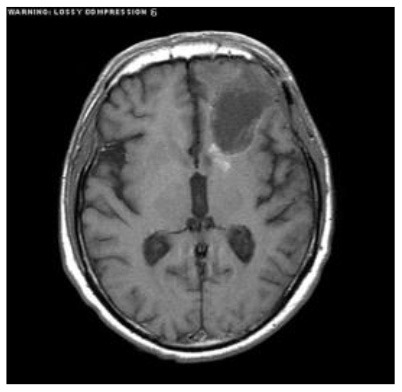	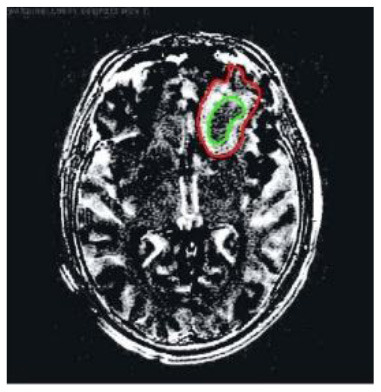
Patient 2	Coronal	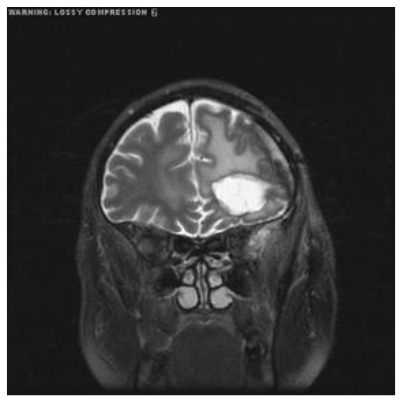	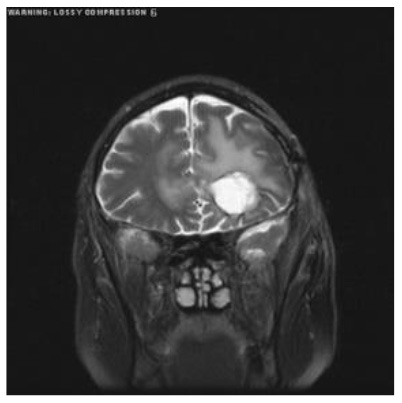	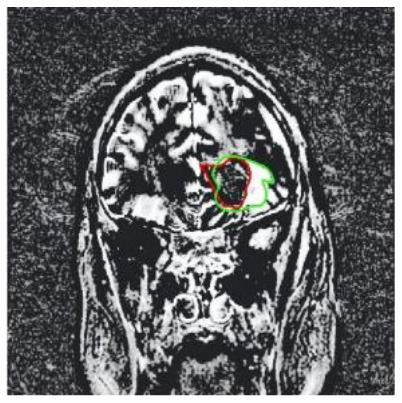
Patient 3	Sagittal	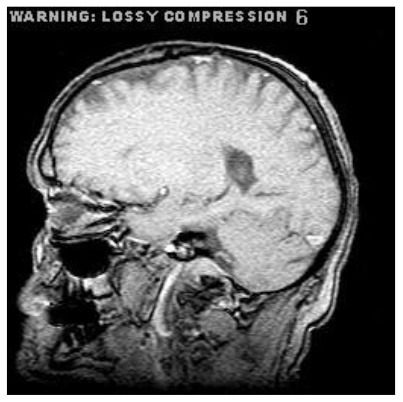	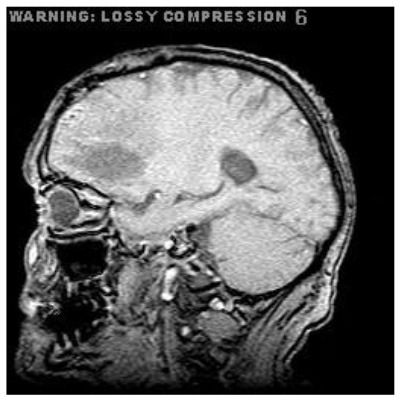	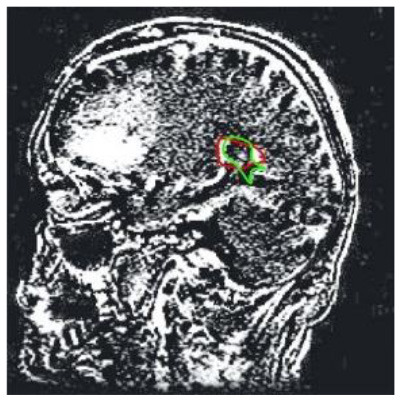
Patient 4	Axial	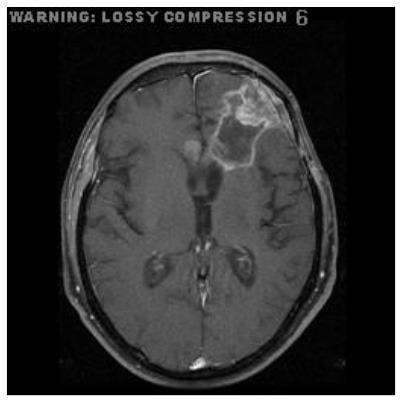	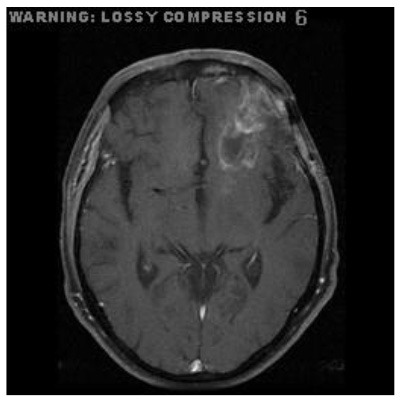	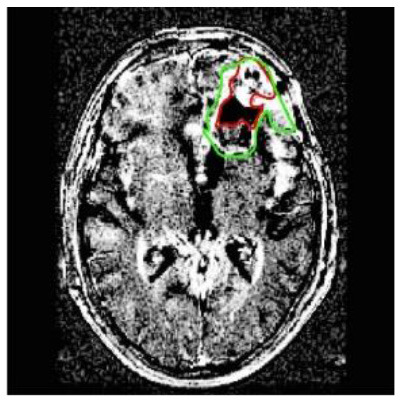
Patient 5	Coronal	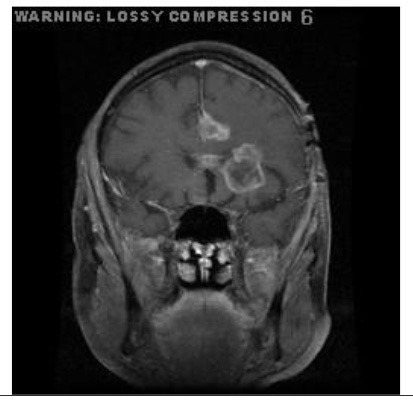	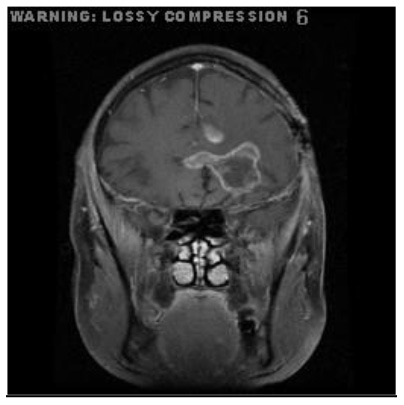	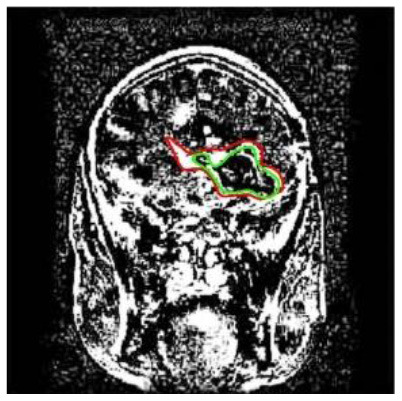

Patient 5 is a radiation necrosis (RN) patient with malignant rhabdoid and a posttreatment entity (43). It occurred 3 months after radiotherapy and gave the effect of capillary damage. Patient 4 demonstrated a pseudoresponse after receiving bevacizumab for the past 7 months. Axial, coronal and sagittal slices of brain MRI sequences of original and current images as well as the subtracted image were shown. These result slices of registration subtract images are colour coded to label signal changes (volume and diameter) over time. Green denotes new or progressive tumours, and red denotes the original tumour.

#### Tumour Diameter Analysis

Table 3 indicates that the auto-subtracted registration diameter of the tumour is close to the gold standard manual conventional registration diameter, with patients 1 and 5 correctly identified to show progression in the size of abnormalities. Diameters of current images **t**_**2**_ decrease (Patient 1, Δ **t**_**2**_ = −0.42 and Patient 5, Δ **t**_**2**_= −0.22) from manual conventional registration to autosubtract registration increase in original images **t**_**1**_ (Patient 1, Δ **t**_**1**_ = 0.04 and Patient 5, Δ **t**_**1**_ = 0.02) for both cases. Abnormality diameters for patients 2, 3 and 4 in both current **t**_**2**_ and original **t**_**1**_ images (Δ **t**_**2**_ = −0.34, −0.51, −0.55 and Δ **t**_**1**_ = −0.49, −0.14, −0.78) decrease when the autosubtract registration technique is used. This finding revealed the precision of autosubtract registration reading. Interreader agreement was high at κ = 0.92 for autosubtraction reading, whilst that of manual conventional registration reading was κ = 0.78.

**Table 3:** Diameter difference of tumour between manual conventional reading and digital image subtraction technique.

**Patient**	**Manual conventional registration diameter (Abnormality progression)**		**Autosubtract registration diameter (Abnormality progression)**	
	**Current Image, *t*_2_ (cm)**	**Original Image, *t*_1_ (cm)**	**Current Image, *t*_2_ (cm)**	**Original Image, *t*_1_ (cm)**
1	3.34	4.43	2.92	4.47
2	3.45	3.34	3.11	2.85
3	3.03	2.43	2.52	2.29
4	4.58	2.91	4.03	2.13
5	1.00	1.04	1.68	1.06

Table 4 presents the comparison of tumour volume between the use of conventional reading and implementing the digital image subtraction method to determine the brain tumour progression using Equation (8). The tumour volume for conventional manual reading and digital image subtraction technique is 3.03 (±22.51) and 2.09 (±22.41) *cm*^3^, respectively. These results showed that the similarity of tumour volume between both methods verifies the precision of autosubtract registration reading. Inter-reader agreement for autosubtraction reading was high at κ = 0.92, whilst that of manual conventional registration reading was κ = 0.78. The tumour volume can be calculated as Equation 8, which is the standard ellipsoid formula. An ellipsoid is the closed surface of which all plane cross-sections are ellipses or circles, which symmetrically intersects around three mutually perpendicular axes in the middle.


(8) $$\begin{array}{l} \text{Tumorvolume }=\text{ length }\times \text{width }\times \text{height }\times 0.52÷\;\frac{\pi }{6} \end{array}$$

**Table 4:** Comparison of tumour volume between manual conventional reading and digital image subtraction technique that determines the brain tumour progression.

**Patient**	**Manual conventional registration (*cm*** ^**3**^ **)**		**Autosubtract registration (*cm*** ^**3**^ **)**		**Brain tumour progression** (***V***_*****t***2**_ **– *V***_*****t***1**_**) (*cm^3^*)**	
	***V*_*t*1_**	***V*_*t*2_**	***V*_*t*1_**	***V*_*t*2_**	***V*_*manual*_**	***V*_*digital*_**
1	45.52	19.51	46.77	13.04	−26.01	−33.73
2	19.44	21.56	12.12	15.75	2.12	3.63
3	7.51	14.57	6.29	8.38	7.06	2.09
4	12.90	50.30	5.06	34.27	37.4	29.21
5	0.59	3.62	0.62	2.48	3.03	1.86

#### Reading Time and Diagnostic Confidence

Compared with manual conventional evaluation based on both current and original images (manual 2.16 ± 0.38 min, auto: 0.77 ± 0.08, *p* < 0.0001), the time required for autosubtract registration reading was substantially lower. Meanwhile, the diagnostic confidence with autosubtract registration reading was graded essentially higher (*p* < 0.0001) ( **t**_**1**_: 1.63 ± 0.58, **t**_**2**_: 1.27 ± 0.45) compared with that of manual conventional reading ( **t**_**1**_: 1.92 ± 0.39, **t**_**2**_: 1.98 ± 0.41).

### Qualitative Assessment

The validated results of precision, accuracy and the time taken to detect interval changes were compared with the manually obtained results. Observers rated an accuracy of 70% in detecting the tumour and abnormalities in the five subtracted images. The overall quality of subtracted brain images in all five patients in terms of precision, accuracy and time taken to detect interval changes is nearly the same at Kappa, κ = 0.83.

### Quantitative Assessment

Table 5 summarises the quantitative errors of transformation parameters. RMSE, which varies from 0.13 to 1.03 mm for translation, is equal in magnitude to ~0–4 pixels for brain MRI and subtracted images. RMSE can be expressed as follows:


(9) $$\begin{array}{l} \text{RMSE}=\sqrt{\frac{\sum_{i=1}^{n}{\left(P_{i}-O_{i}\right)}^{2}}{n}} \end{array}$$

where *P* is the predicted value of the image translation, *O* is the observed value of the image translation with image variables and *i* and *n* indicate the number of sample images.

**Table 5:** RMSE of transformation parameters of the brain MRI.

**Patient**	**Interval changes translation (mm)**	
	**Δtx**	**Δty**
1	0.41	0.13
2	0.48	0.20
3	1.03	0.93
4	0.82	0.32
5	0.41	0.15

*Δtx and Δty refer to RMSE for the translation*.

## Discussion

Five tested brain images presented interval changes due to various abnormalities. The image registration was very successful for these pairs. Subtraction images can enhance the clarity of various interval changes, including the change in size, shape, and location of abnormality masses in the brain area. The digital image subtraction technique also clearly increased the confidence of observers due to the absence of clinically significant interval changes in other regions. An interdisciplinary tumour board along with neuro-radiologists, neurosurgeons, neurologists and radiation oncologists also use this approach to identify various potential forms of diseases that affect the brain organ by applying the detailed criterion behaviour of abnormality (44).

The error of Δ*tx* and Δ*ty* translation was calculated using RMSE from both registration methods shown in Table 5. A computerised scheme for the analysis of digitised medical images provides the enhancement of interval changes that occur in a pair of temporally sequential images using a pair of autodigitised images in the method of invention. The image pair is then subjected to image registration, including a linear warping of one of the images to ensure that corresponding locations in the two images are aligned with each other. The subtraction process can be performed after image registration to generate the difference between the original and current images. In this manner, slight opacities present in current images can be detected on the basis of the subtraction of the registered brain MRIs.

Image subtraction is necessary for diagnosing patients with brain cancer, even though it is rarely used. This technique is intended to remove undesirable and distracting shadows, has long been employed in conventional radiography as a visual assistant. It is not using a complicated algorithm, which is a very straightforward algorithm, and it is easy to train new physicians to understand the monitoring for a brain tumour. It is digitally removed unenhanced images from enhanced images that are beneficial for MR imaging (45). The augmentation of contrast increases the diagnostic sensitivity and specificity of brain MR imaging (46). This approach can improve the delineation of the pattern and degree of enhancement of brain tumours by reducing the time required for diagnosis and preventing tumours from migrating to other organs. According to Equation (9), high RMSE results indicate a large difference between reference and target images. Meanwhile, a low RMSE value indicates improved image production (47). The registration and subtraction results showed minimal distance errors in images. The framework of this image subtraction technique on brain images provides the diagnosis precision and reduces the workload of physicians involved in imaging analysis (48).

This study analysed an OPC UA-certified and FDA-approved MATLAB software framework to generate and read digital image subtracts for brain tumour imaging based on pairs of temporally different images (49, 50). The evaluation of a solid tumour by different observers can vary; for example, observers can select varying measurement parameters and result in an uncertain measurement of the diameter of an irregularly shaped abnormality. Some observers may include the cystic area whilst others may ignore it. Even the use of volume as a parameter for acquiring accurate measurements is challenging. Providing diagnostics with the maximum degree of precision has become increasingly complicated for radiologist with the growing case numbers. However, the use of the proposed method can achieve accurate measurement and advance digital image technology.

Automatic co-registration and contrast coding are important benefits of the proposed approach over the conventional manual method. The key results were improved sensitivity, precision, and diagnostic confidence for auto-subtracted registration reading along with factually decreasing scan times and high spatial resolution for image evaluation relative to conventional manual reading. We must note that dedicated neuro-radiologists that use large neuro-oncological caseloads at a specialist academic centre performed studies with similar sequence parameters for current and original images and obtained nearly perfect (no false positive and negative results) diagnoses (51) for auto-subtracting registration readings, thereby optimising the implementation of the digital image subtraction technique. However, the automated image subtraction algorithm can outperform the standard manual method given these situations.

The following limitations have been recognised in the evaluation of outcomes in this study. Firstly, the volume was directly calculated without considering the actual volume of the tumour using the metric of tumour diameter. Secondly, the lack of comparative data that limits the analysis is a serious obstacle. A large size of image samples and a substantial relationship between both techniques are needed to determine the pattern. Although the time taken for the subtracted registration reading was substantially lower than that of the standard manual method reading, the time required for the digital image subtraction technique must also be determined. However, with little effort, direct automated routing of image sequences from the MRI scanner to the application of this technique's development seems practicable, ideally complemented by the fully automatic initiation of the generation of image subtraction techniques during the clinical routine. Additional data are needed to test this algorithm in the future. Additional observers must be included in the routine medical imaging diagnosis to obtain advanced information. Different observers exhibit varying cognitive, visual and perceptual expertises in understanding the performance of specific medical imaging techniques.

## Conclusion

The digital image subtraction technique plays a crucial role in assisting physicians in diagnosing diseases via medical imaging, such as brain MRIs. The proposed work will incorporate high diagnostic strength and image guidance procedure in brain MRI for enhanced accuracy in treatment plan. Furthermore, we should be able to track abnormalities easily and rapidly with high degree of accuracy. MATLAB software can show the accuracy of image data in image subtraction. This pilot study proves the effectiveness of using the digital image subtraction technique in detecting the progress of brain tumour disease.

Lastly, the integration of machine learning and magnetic radiomic functionality with image subtraction analyses is important in enhancing the efficacy of treatment-related results on tumour recurrence in future neuro-oncological imaging research. The performance of the automated image subtraction method may be further improved. Physicians who took part in the interpretation diagnosis now just got guidance on the temporal image subtraction with only more <15 min preceding understanding. Physicians can improve their skills in temporal image subtraction and surpass their daily caseload limit in their clinical routine with the proposed technique. Hence, a progressively viable temporal and spatial image subtraction diagnosis can be established in future investigations. High-performance technology is essential for patients for their safe and effective diagnostic examinations.

## Data Availability Statement

Data used to support the findings of this study are available from the corresponding author upon request.

## Ethics Statement

This retrospective study was performed after obtaining prior approval from the Medical Research and Ethics Committee (MREC) and the ethics committee of Universiti Sains Islam Malaysia (USIM).

## Author Contributions

All authors listed have made a substantial, direct and intellectual contribution to the work, and approved it for publication.

## Funding

This study was financially supported by the Universiti Sains Islam Malaysia Research Grant (PPPI/FST/0119/051000/17519).

## Conflict of Interest

The authors declare that the research was conducted in the absence of any commercial or financial relationships that could be construed as a potential conflict of interest.

## Publisher's Note

All claims expressed in this article are solely those of the authors and do not necessarily represent those of their affiliated organizations, or those of the publisher, the editors and the reviewers. Any product that may be evaluated in this article, or claim that may be made by its manufacturer, is not guaranteed or endorsed by the publisher.
